# Beyond Antioxidants: The Microbial Metabolic Landscape of Anthocyanins and Their Downstream Health Implications

**DOI:** 10.3390/nu18091413

**Published:** 2026-04-29

**Authors:** Yan Zeng, Munir Ahmed, Hua Zhang

**Affiliations:** Department of Food Nutrition and Safety, College of Pharmacy, Jiangxi University of Chinese Medicine, Nanchang 330004, China; zengyan2@jxutcm.edu.cn (Y.Z.); munirahmed@jxutcm.edu.cn (M.A.)

**Keywords:** anthocyanins, gut microbiota, microbial metabolites

## Abstract

Background/Objectives: Anthocyanins are dietary pigments associated with reduced risk of chronic diseases, yet their low systemic bioavailability challenges the traditional direct antioxidant hypothesis. This review aims to reconceptualize anthocyanin bioactivity by proposing the gut microbiome as a key mediator that biotransforms these compounds into bioactive metabolites responsible for systemic health effects. Methods: This review synthesizes evidence on the microbial metabolism of anthocyanins and includes a structured appraisal of the literature using an evidence evaluation framework analogous to GRADE, focusing on their transit to the colon, enzymatic biotransformation by gut microbiota, and resulting production of phenolic metabolites such as protocatechuic acid (PCA). It also examines the role of specific bacterial taxa (e.g., *Bifidobacterium* and *Lactobacillus*) in enhancing bioavailability and explores the downstream cellular pathways modulated by these metabolites. Results: Gut microbiota convert anthocyanins into smaller phenolic metabolites such as PCA, syringic acid, gallic acid, and other respective metabolites, which achieve plasma concentrations up to 100-fold higher than parent compounds and can cross the blood–brain barrier. These metabolites exert systemic effects by modulating key signaling pathways (NF-κB and Nrf2) and restoring redox homeostasis. Additionally, beneficial gut bacteria enhance anthocyanin bioavailability and support the production of short-chain fatty acids (SCFAs). Conclusions: Systemic health benefits of anthocyanins are largely mediated by gut microbiota through the generation of bioactive metabolites. This microbiota-driven process redefines the mechanistic understanding of anthocyanin action and highlights the microbiome as a critical determinant of their efficacy in preventing cardiometabolic and neurodegenerative diseases.

## 1. Introduction

Anthocyanins are pigment-containing, water-soluble substances in plants that produce identical colors of red, purple, or blue (pigment color profiles) and are associated with a variety of health benefits [1]. Substantial epidemiological and clinical evidence demonstrates that a high dietary anthocyanin intake (from berries, grapes, cherries, etc.) could help to reduce the risk of developing chronic diseases (e.g., heart diseases, type 2 diabetes, neurodegenerative diseases, some cancers, etc.) [2]. This has generated considerable interest in their underlying mechanisms. On the other hand, the ‘anthocyanin paradox’ demonstrates the enormous discrepancy between health effects and the very low systemic bioavailability of these compounds [3]. After being consumed, only <5% of anthocyanins are absorbed intact because of the poor conditions in the gastrointestinal tract, particularly regarding changes in pH and the actions of digestive enzymes [3,4], resulting in plasma concentrations in the nanomolar range that are far below the levels required for direct pharmacological effects in vitro [5]. Given the limited systemic absorption of parent anthocyanins, it is important to reconsider how dietary anthocyanins provide their health benefits.

Historically, the health-promoting properties of anthocyanins have generally been attributed to their direct antioxidant properties and their ability to scavenge free radicals [6]. While in vitro studies of anthocyanins have demonstrated strong antioxidant activity [7], the in vivo relevance of this antioxidant hypothesis is under considerable scrutiny [8]. Since intact anthocyanins do not get into plasma or target tissues in concentrations as high as the body’s antioxidant system (e.g., glutathione and uric acid), it is unlikely that they function as direct free radical scavengers in vivo [9,10]. Consequently, most of the research has centered around indirect pathways. Specifically, it is thought that anthocyanins may modulate core signaling pathways, which will enhance some antioxidant enzyme activities (for example, SOD, catalase), as well as inhibit some pro-inflammatory pathways (e.g., NF-κB) [11,12]. This mechanistic shift reveals an important missing piece in understanding how the parent compound itself does not directly cause biological activity. As such, it would seem probable that there are large numbers of systemically absorbed microbial metabolites present within the body that actually provide the health benefits observed.

A growing consensus indicates that the gut microbiome serves as a complex ‘metabolic organ’ composed of trillions of microbial communities, and it acts as a critical and largely neglected biological mediator of anthocyanin bioactivity [13]. Because a significant portion of anthocyanins reaches the colon unabsorbed, they provide a substantial substrate for resident microbiota. Through diverse enzymatic processes, including beta-glucosidases and decarboxylases, the microbiota biotransforms complex glycosylated parent structures into smaller, more absorbable, and often more biologically active non-anthocyanin derivatives such as phenolic acids (e.g., vanillic and protocatechuic acid) [14,15]. These colonic metabolites, rather than the parent compounds, accumulate systemically and can even cross the blood–brain barrier to regulate local biological activities [16]. This interaction is bidirectional: anthocyanins exert prebiotic effects, selectively modulating microbial diversity and functional output [17,18]. Interventions have been shown to ameliorate diet-induced dysbiosis by promoting beneficial bacteria like *Bifidobacterium* and *Lactobacillus*, while increasing the production of short-chain fatty acids (SCFAs) like butyrate and propionate [18,19]. These SCFAs exert profound effects on host metabolism, immunity, and gut barrier integrity [20]. Further, recent studies increasingly support this concept. The health benefits of anthocyanin compounds do not solely rely on their availability in blood (although studies show this); rather, their bioactive metabolites interact with hosts and microbial systems. The growing concept of anthocyanins acting as a “prodrug” refers to the idea that their health benefits are not primarily due to the intact anthocyanin molecules themselves, but rather to the bioactive metabolites produced after their transformation by the gut microbiota [21]. In essence, classifying anthocyanins as a “prodrug” emphasizes that their therapeutic potential is largely unleashed after microbial processing in the gut, making the gut microbiota a critical determinant of their ultimate health impact [22].

Consequently, this review proposes that the gut microbiome plays a key role in mediating the health effects of anthocyanins and that the historical understanding of anthocyanins is lacking; instead, the gut–microbiome–metabolite axis can be considered a central framework for understanding anthocyanin-mediated health effects. By systematically evaluating current literature, the therapeutic effect of anthocyanins is evaluated through a two-prong approach: on the one hand, microbial conversion of anthocyanins yields bioactive metabolites; on the other hand, anthocyanins modulate the gut environment in a manner consistent with prebiotics, creating a paradigm shift to consider the microbiome as the unifying center for chronic disease prevention.

## 2. Methodology

This review uses a structured literature search and thematic synthesis. We retrieved relevant publications from PubMed, Web of Science, ScienceDirect, and Google Scholar, covering publications from 2019 to 2025, to provide an accurate representation of the most recent advances in and current knowledge of the metabolism of anthocyanins by the gut microbiota. In addition, earlier key studies were included where necessary to provide foundational insights. At the start of the search, keyword combinations such as “anthocyanins,” “gut microbiota,” “microbial metabolism,” “phenolic metabolites,” “bioavailability,” and “health benefits” were used to identify articles published within the 7 years that analyzed anthocyanin metabolism, gut microbiome interactions, and related health outcomes. In addition to using keyword searches, additional studies were identified through the review and screening of the reference lists of relevant reviews and primary research articles. Relevant publications were screened based on title, abstract, and full-text evaluation to present a conceptual and mechanistic understanding of the anthocyanin–microbiome–metabolite axis. We excluded the non-English papers, conference abstracts without complete experimental data, and studies not directly relevant to the scope of this review.

## 3. Journey of Anthocyanins in the Gastrointestinal Tract

The pharmacokinetics of anthocyanins involves a complex series of biotransformation and absorption events occurring throughout the gastrointestinal tract (GIT). Native anthocyanins are poorly absorbed (<5%); therefore, their biological effects are largely mediated within the GIT [15,23]. Although the upper gastrointestinal tract generally absorbs small amounts of intact glycosides by way of passive diffusion and transporters such as SGLT1 [5], their stability is dependent on specific structural characteristics; for example, polyglycosylic or acylated versions are more resistant to breakdown than their monoglucosidic version during their passage through the upper gastrointestinal tract [24]. Even so, most dietary anthocyanins are not absorbed in the upper part of the gastrointestinal tract. Therefore, they travel to the distal gut and become substrates for microbiota [15]. As a result, the distal gastrointestinal tract is considered a critical site for microbial interactions that support systemic health benefits of anthocyanins.

The colon represents a key site where unabsorbed anthocyanins undergo extensive microbial metabolism, accounting for approximately 90% of the total initial anthocyanin dose [25]. Anthocyanins enter the colon in a highly concentrated form because they have been associated with dietary fibers within the food matrix. As a result, they are now considered potential nutrients that might be absorbed in the host and act as substrates for the complexly active colonic microbiota [15]. Once in the colon, the food matrix continues to interact with gut microbiota. The interaction between dietary components and gut microbiota influences anthocyanin metabolism. Thus, the availability of fermentable dietary fiber consumed daily through digestion and its fermentation to produce short-chain fatty acids (SCFAs) is a key factor that influences the gut microbiota environment [26]. Additionally, food macronutrients and polyphenols will also arrive in the colon along with anthocyanins in the food matrix and will interact and potentially alter the solubility, enzymic release, and mixed micelle partitioning of these compounds, allowing for varying degrees of absorption and microbial biotransformation [27]. Such interactions may be competitive or synergistic and yield distinct profiles of anthocyanin-derived metabolites [28,29].

The use of purified anthocyanins in human intervention studies has its limitations compared to the more complex ‘real life’ situation with food consumption. The metabolism of anthocyanins is highly dependent on the food product/matrix, daily anthocyanins intake, and individual gut microorganisms [30,31]. Future studies would benefit from a whole-food approach as well as the use of more physiologically relevant model systems to study the potential health benefits of consumption of anthocyanins and the interplay between the gut microbiome and these food metabolites.

The flavylium cation form of anthocyanins, which maintains its relative stability in the low-pH environment of the stomach (pH 1.5–3.0), undergoes significant destabilization upon exiting the stomach and entering the near-neutral environment of the small intestine (pH 6.0–7.5), leading to structural instability of the flavonoid backbone and the formation of hemiketal and quinoidal structures [32]. The structural changes make them vulnerable to hydrolysis, ring splitting, and oxidation, which destroy the flavonoid skeleton. This structural instability facilitates microbial utilization of unabsorbed anthocyanins as a key source of carbon by resident microorganisms such as *Bifidobacterium* and *Lactobacillus* [14]. The metabolic utilization of these available anthocyanins is initiated by bacterial deglycosylation via bacterial enzymes, including β-glucosidase, which removes the sugar portion of the anthocyanins, producing the corresponding aglycone compounds [33]. In contrast to their well-characterized stable glycosylated parent compounds, aglycones are highly labile in the near-neutral pH environment of the colon and are rapidly subjected to further catabolism, particularly through C-ring cleavage [34]. Ring cleavage breaks down the structure of the anthocyanins into smaller molecules, producing phenolic acids such as protocatechuic acid, vanillic acid, and phloroglucinol derivatives. These compounds have higher bioavailability when absorbed by humans than the original compounds [35]. Ultimately, these metabolites that are produced through the actions of microorganisms will be detected in systemic circulation or urine, indicating how much the host’s gut microbiome plays a role in determining the systemic bioactivity of anthocyanins and their impact on redox signaling [36]. The interaction between anthocyanins and gut microbiota is bidirectional and an important symbiotic relationship that plays a critical role in health outcomes and well-being. There are two key mechanisms involved with this relationship: (1) the selective stimulation of beneficial bacteria and (2) the suppression of pathogenic bacteria; these mechanisms will contribute to a prebiotic type of environment in the gut [37].

## 4. The Chemical Basis of Anthocyanin Metabolism by Gut Microbes

Anthocyanins are structured around a central flavylium cation skeleton. Since the structure of anthocyanin determines their metabolic fate, it is important to consider the central backbone and the positions at which substituents are attached, giving rise to different anthocyanidins, such as cyanidin or malvidin. Based on the basic framework, the backbone is two benzyl rings (A and B) that are linked to a three-carbon heterocyclic ring (C) (Table 1). The chemical structure formula is C_15_H_11_O_6_ + R1 and R2. These are most often put on the 3- and 5-positions of the B-ring. They are usually hydrogen (-H), hydroxyl groups (-OH), or methoxyl groups (-OCH_3_). The color is defined by the B-ring, and the magic of stability occurs at the position in the C-ring by the term R3 (Table 1). Naturally, an anthocyanidin (the aglycone) is highly unstable until a sugar molecule is conjugated at that 3-position, making it stable enough to be a true anthocyanin [37]. Glycosylation significantly enhances anthocyanin solubility and provides a critical defense against light and enzymatic degradation. The addition of glucose moieties, particularly through di-glycosylation at the end positions, not only improves water solubility but also substantially increases the molecule’s structural stability [38].

Furthermore, the conjugation of these sugars with acyl groups (such as p-coumaric, ferulic, or malonic acid) provides an optimal stability profile. Specifically, the addition of a second sugar at the 5-position serves as a protective buffer against fluctuations, rendering these acylated and polyglycosylated derivatives far more resistant to thermal and photo-degradation than their simpler counterparts. That is why red cabbage, for example, retains its characteristic purple hue even during cooking. The R3 position plays a pivotal role in the ‘anthocyanin paradox’. The sugar moiety attached to this site acts as the primary gateway for biological processing, as demonstrated in Table 2. Although human transport proteins, such as SGLT1, recognize these specific sugars (typically glucose) to facilitate initial cellular uptake, the human body lacks the endogenous enzymes (such as specialized β-glucosidases) necessary to cleave the bond at the position. This enzymatic deficiency ensures that the anthocyanin remains largely intact until it reaches the microbial environment of the lower gastrointestinal tract.

## 5. Biotransformation of Anthocyanin by Gut Microbiota

**Figure 1:**
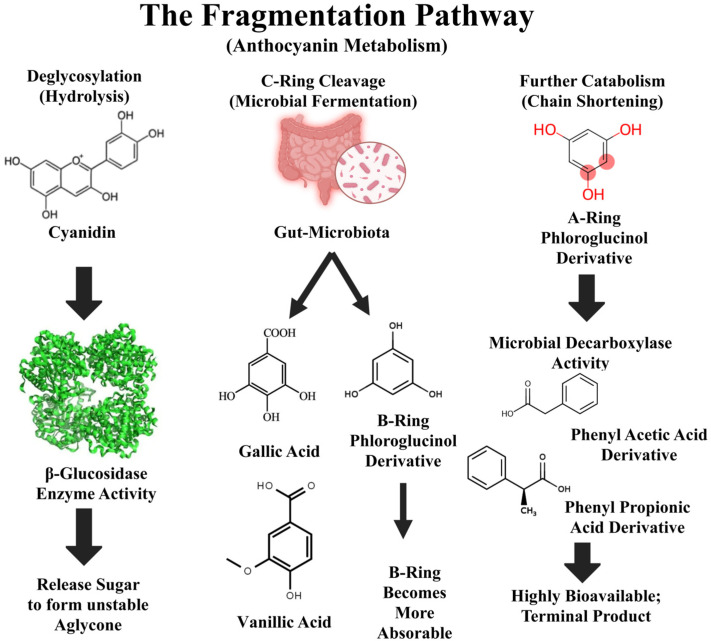
Fragmentation Pathway of Anthocyanin Metabolism in Gastrointestinal Tract. This diagram illustrates the multi-stage microbial biotransformation of anthocyanins, including deglycosylation by β-glucosidases, formation of aglycones, subsequent C-ring cleavage, and generation of phenolic metabolites such as gallic acid, vanillic acid, and phenylacetic acid derivatives. Created in BioRender. Ahmed, M. (2026) https://BioRender.com/p9farv8.

The concept of anthocyanins as effective health agents is fundamentally redefined by the metabolic machinery of the gut microbiome [14]. Since the bulk of parent compounds survive the upper digestive tract, their true therapeutic potential is unlocked through biotransformation into smaller, highly bioavailable phenolic acids and simple phenols [4]. The resulting shift in focus from the direct antioxidant capacity of the parent molecule to the biological activity of its microbial-derived metabolites is the central paradigm change motivating this review. The catabolism of anthocyanins by the colonic microbiota is a multi-step process, predominantly carried out by specialized bacteria such as *Bifidobacterium* spp. and *Lactobacillus* spp. [39]. This process systematically dismantles the large, complex flavonoid structures into smaller absorbable derivatives [40]. The typical fragmentation pathway is shown in Figure 1. Based on this pathway, it is suggested that the anthocyanin–microbiome–metabolite axis may represent a potential target for improving systemic health.

### 5.1. Deglycosylation

The initial step in the microbial metabolism of anthocyanins is the cleavage of the O-glycosidic bond that links the sugar moiety (e.g., glucose or rutinose) to the core anthocyanidin structure [35]. This hydrolysis reaction is catalyzed by bacterial enzymes such as β-glucosidase, which converts anthocyanin–glycosides to their corresponding aglycones [15]. This transformation represents a critical metabolic step because the parent glycosides are relatively stable, whereas the resulting aglycones (e.g., cyanidin, delphinidin, and malvidin) are highly unstable in colonic pH conditions [41]. Consequently, these aglycones are subjected to sequential microbial degradation in the gut. A summary of the gut microflora-derived metabolites and their effects on health is provided in Table 3. Accumulating evidence indicates that these secondary metabolites, rather than the parent compounds or intermediate aglycons, primarily contribute to systemic disease management through the observed clinical benefits, such as antioxidant, anti-inflammatory, neuroprotective, and cardiovascular benefits [42]. Composition and diversity of the metabolites depend on the functional capacity of the gut microbiome, further emphasizing the central role of microbial metabolism in anthocyanin bioactivity.

### 5.2. Ring Fission: The Gatekeeper of Anthocyanin Bioavailability

**Figure 2:**
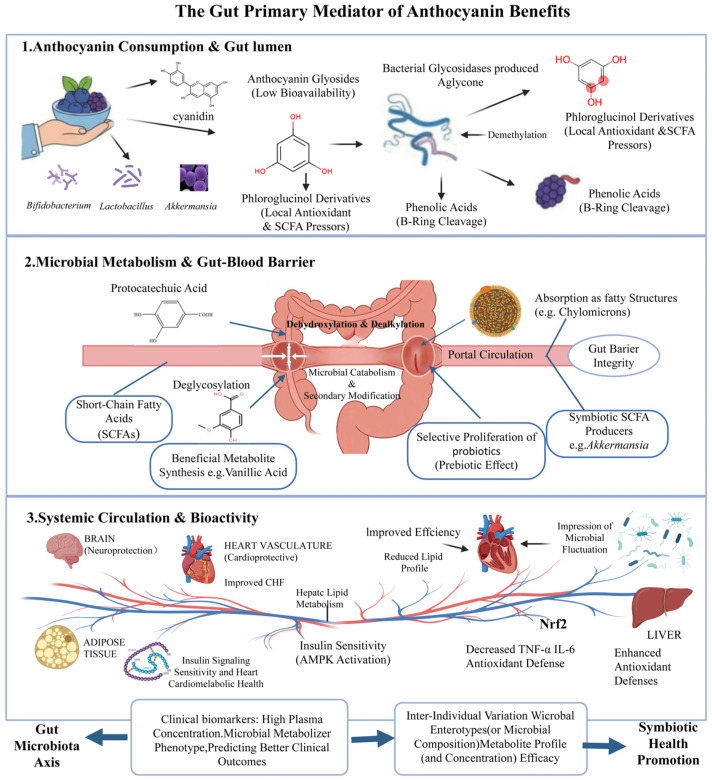
Gut–Microbiota–Metabolite Axis in Anthocyanin Bioactivity. This schematic diagram illustrates the microbial conversion of dietary anthocyanins into intermediate compounds through deglycosylation and ring fission, followed by further metabolism into phenolic acids and short-chain fatty acids. The diagram also shows the association of these metabolites with microbial composition and systemic distribution. Created in BioRender. Ahmed, M. (2026) https://biorender.com/h3t097u.

Following the deglycosylation, the unstable anthocyanidin aglycon undergoes heterocyclic C-ring fission, a transformational reaction that disrupts the core flavonoid skeleton. This metabolic transformation occurs in sequential stages, which are mediated by gut microbiota such as *Clostridium* spp., *Eubacterium* spp., and *Bacteroides* spp. [49,50]. Initially, cleavage of the A-ring within the benzopyrylium skeleton produces smaller, less complex phenolic compounds (mostly phloroglucinol derivatives) [38]. A-ring cleavage is considered a rate-limiting step in all future microbial degradation processes. Once the A-ring has been cleaved, the C-ring (which contains oxygen) also breaks down, leading to the cleavage of the C-3 chain [13]. This degradation process gives rise to short-chain phenolic acids that serve as substrates for further microbial transformation. After the A-ring is cleaved, phloroglucinol and its derivatives (e.g., phloroglucinaldehyde) are produced. Phloroglucinol derivatives serve as potent local redox signaling agents to protect the epithelial cells from oxidative damage and to promote optimum colonization of the gut by the host and beneficial colonic bacteria [51]. Phloroglucinol A and C-ring fragments also create selective pressure on beneficial anaerobic bacteria like Bifidobacteria and *Faecalibacterium prausnitzii*, which can use these newly formed substrates due to their production of appropriate enzymes [41]. Additionally, fragments of the A-ring of anthocyanins, via further bacterial metabolism, contribute to the pool of substrates that can be utilized to produce the SCFAs acetate, propionate, and butyrate [14]. These transformations generate bioavailable phenolic metabolites that enter systemic circulation, and underline the therapeutic potential of anthocyanin-rich diets in managing chronic conditions, including type 2 diabetes and cardiovascular disorders [52]. Taken together, by sharing the initial steps of C-ring fission, anthocyanin intake provides a dual-functional role, as shown in Figure 2, including delivering low-molecular-weight phenolic acids into circulation for antioxidant action in the brain and vasculature [37,43], and strengthening the intestinal barrier and enhancing SCFA production through the provision of substrates and local antioxidant protection [21].

### 5.3. Subsequent Reactions: Secondary Biotransformation

Subsequent biotransformation includes dehydroxylation, demethylation, and decarboxylation reactions, which convert complex intermediates into simple phenolic compounds [53]. The physiological significance of these reactions stems from their ability to enhance the aqueous solubility and lipid permeability of metabolites. These modifications enhance solubility and membrane permeability, thereby improving systemic absorption and bio-efficacy.

A key aspect of the secondary metabolism is demethylation, involving the removal of the methyl group (-CH_3_) from methoxylated phenolic acids (e.g., malvidin, peonidin, petunidin) [35]. The primary functions of demethylation are shown in Figure 2. Demethylation converts less active methoxylated compounds into active hydroxylated derivatives. For instance, vanillic acid (methoxylated) is changed to protocatechuic acid (PCA) (hydroxylated) through demethylation [36]. Another function is that hydroxylated compounds are much stronger antioxidants than methoxylated compounds. Free hydroxyl (-OH) groups offer extra free radical scavenging ability [54]. Demethylation also increases metabolite solubility, facilitating systemic absorption and thus determining an increased rate of absorption and achieving a higher percentage of absorption [55].

Demethylating microorganisms, including strains from the *Clostridium* genus, are indicative of an efficient microbiome that metabolizes anthocyanins [56]. Dehydroxylation, or the removal of a hydroxyl (-OH) group, results in molecules that contain a lesser degree of polarity (more lipophilicity) [17,57]. The primary reason for dehydroxylation is to convert phenolic compound metabolites into hydrophobic (lipophilic) forms that more efficiently diffuse through the lipid bilayer of colonocytes and enter the portal circulation, therefore increasing systemic absorption efficiency [58]. For example, dehydroxylation of PCA can lead to the formation of coumaric acid [36]. These less hydroxylated compounds may exhibit different bioactivities, allowing the anthocyanin pathway to generate a broader spectrum of bioactive molecules [49].

Dehydroxylation-produced metabolites are frequently present in much higher concentrations in peripheral tissues and play a key role in mediating systemic effects such as neuroprotection and cardiovascular benefits [59]. Decarboxylation involves the detachment of the carboxyl group -COOH, yielding simple phenols. Decarboxylation produces small, simple, phenolic compounds like phloroglucinol and catechol, which are low molecular weight and will be readily absorbed [15]. These compounds produce a rapid but transient increase in bioavailability; they are eliminated rapidly and therefore cause the immediate acute effects experienced following anthocyanin intake [60]. This is often the last step in the complete degradation pathway for aromatic rings by microbes [36]. The simple phenolic compounds will remain stable in the bloodstream and will be efficiently conjugated with glucuronides or sulfates in the liver before being removed from the body via urine [61]. There are certain bacterial enzymes, such as phenylacrylic acid decarboxylase, that enable the microbiota to decarboxylate various compounds. This exemplifies the metabolic latitudes available to process and handle the wide variety of dietary polyphenols [56].

These secondary modifications are essential for converting unstable intermediates into bioactive metabolites. The small, lipid-soluble phenolic compounds created as a consequence of microbial transformation are considered key contributors to the systemic effects of redox signaling and immunomodulatory effects seen [62]. These sequential microbial transformations generate a diverse pool of phenolic metabolites, which form the basis of the systemic metabolic profile discussed in the following section.

## 6. Microbial Metabolic Profiles and Their Biosynthetic Pathways

**Figure 3:**
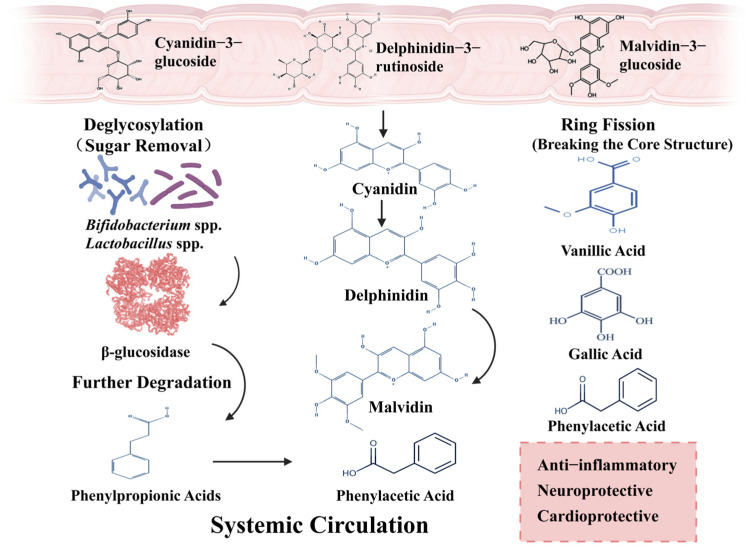
Anthocyanin–Microbiome Metabolic Cascade: Biotransformation into Systemically Active Phenolic Acids. This figure depicts the degradation of anthocyanins by gut microbiota into smaller phenolic compounds following deglycosylation and ring cleavage. These metabolites are transported into systemic circulation and distributed to peripheral tissues. Created in BioRender. Ahmed, M. (2026) https://BioRender.com/g31l6uz.

The microbial metabolism of anthocyanins generates a diverse spectrum of low-molecular-weight phenolic compounds, each characterized by distinct structural features and biological functions [37]. The degradation of the anthocyanidin B-ring generates a variety of phenolic derivatives (Figure 3), which include terminal metabolites from microbial fermentation [4,56]. Low-molecular-weight compounds produced from decarboxylation or reduced in length from initial phenolic acids are well absorbed and contribute greatly to the overall systemic polyphenol burden [15]. The metabolic diversity of anthocyanins allows them to impact a wide spectrum of host physiological processes, leading to a variety of health effects seen clinically, as depicted in Figure 3.

### 6.1. PCA

Protocatechuic acid (PCA; 3,4-dihydroxybenzoic acid) is considered a major circulating metabolite consistently observed at higher concentrations than parent anthocyanins in biological systems [63]. PCA is generated through microbial transformation of anthocyanin structures [64]. The formation of protocatechuic acid from anthocyanidins is a multi-step microbial process. Protocatechuic acid (3,4-dihydroxybenzoic acid) is a major and well-characterized catabolite of anthocyanidins, particularly those derived from cyanidin-type anthocyanins, which possess a catechol B-ring (3′,4′-dihydroxyl groups) [15]. Gut microbiota, including species like *Enterobacter cancerogenous*, have been shown to catabolize anthocyanins and produce protocatechuic acid [65].

### 6.2. Gallic Acid

Gallic acid (3,4,5-trihydroxybenzoic acid) is a polyhydroxylated phenolic acid with significant biological activity that represents the principal B-ring degradative product for delphinidin, an anthocyanidin with the deep blue and purple coloration found in blueberries, Concord grapes, and pomegranates [66]. In the gastrointestinal tract, delphinidin undergoes C-ring fission in order to produce gallic acid [67]. The B-ring connects three vicinal hydroxyl groups, providing gallic acid with its significant biological activity. This trihydroxy configuration (three adjacent hydroxyls on the phenolic benzene) results in gallic acid’s well-established radical scavenging effects and may exhibit greater effectiveness at neutralizing reactive oxygen species (ROSs) than the majority of other phenolic acids [68]. Importantly, in comparison to the delphinidin molecule, gallic acid is highly soluble in water, which can be efficiently absorbed from the small intestine and rapidly distributed systemically to distant organs, including the liver and brain. Additionally, gallic acid may be further microbially metabolized by gut microbiota to produce pyrogallol, an additional highly bioactive metabolite [69]. Compared to other phenolic metabolites, the trihydroxy substitution pattern of gallic acid confers enhanced radical scavenging capacity, highlighting the importance of structural hydroxylation in determining antioxidant potential [68].

### 6.3. 4-Hydroxybenzoic Acid

Hydroxybenzoic acid (4-HBA) is classified as monohydroxybenzoic acid and a secondary metabolite detected in human biological systems [70]. In relation to the metabolism of anthocyanins, 4-HBA represents the primary B-ring metabolite resulting from the degradation of the pigment pelargonidin. Pelargonidin contains red-colored pigments that are found in strawberries and raspberries. When pelargonidin undergoes microbial digestion in the gastrointestinal tract, the C-ring is cleaved and released as 4-HBA [71]. The structure of pelargonidin contains one OH group on the B-ring, resulting in only one substituent position on the C-4 location of 4-HBA. In general, this structural simplicity limits the ability of 4-HBA to act as an antioxidant. Lower hydroxyl group (OH) numbers are generally associated with lower radical scavenging abilities than polyhydroxyl phenols such as protocatechuic acid (2 OH groups) or gallic acid (3 OH groups). 4-HBA will typically further be utilized as an intermediate to convert into phenylacetic acid derivatives via bacterial decarboxylation and dehydroxylation; however, it has several reported biological activities [72].

### 6.4. Vanillic Acid

Vanillic acid is primarily formed through the microbial metabolism of anthocyanins via a series of enzymatic biotransformation involving deglycosylation, C-ring cleavage, and subsequent modifications of phenolic intermediates by gut microbiota [73]. It can also be synthesized from protocatechuic acid. PCA is transformed into the vanillate derivative vanillic acid (4-hydroxy-3-methoxybenzoic acid) by microorganisms [74]. Although a direct enzymatic pathway from protocatechuic acid to vanillic acid in the anthocyanin metabolic pathway has not been elucidated, various species of gut microbes, including some *Bifidobacterium* and *Lactobacillus* species and *Eubacterium ramulus*, can convert protocatechuic acid to other methylated or hydroxylated phenolic acids [65]. Furthermore, due to its specific chemical structure, vanillic acid is able to pass through biological membranes easily and can help reduce oxidative stress on neural tissues. In addition, vanillic acid also modulates oxidative stress pathways and contributes to neuroprotective effects through its ability to penetrate biological membranes.

### 6.5. Syringic Acid

Syringic acid (4-hydroxy-3,5-dimethoxybenzoic acid) is a major B-ring metabolite of malvidin, which is the leading type of anthocyanidin found in red wine and fruits with dark skin [75]. Syringic acid (3,5-dimethoxy-4-hydroxybenzoic acid), with its two methoxy groups, generally originates from anthocyanidins with a trihydroxylated B-ring such as delphinidin. The formation of syringic acid is proposed to involve two O-methylation steps on a trihydroxylated benzoic acid intermediate, contrasting with the single methylation seen in vanillic acid formation [76].

These metabolites are considered important because (1) they are systemically bioavailable, and (2) they can be used as clinical biomarkers. These metabolites have the potential to significantly achieve peak plasma levels compared to their intact forms (Figure 3), which further supports that the host’s gut microbiota possesses a functional biosynthetic capacity (i.e., that it possesses all of the necessary enzymatic pathways and bacteria that can metabolize anthocyanins). Because these are very stable and the systemic concentration levels are high, these metabolites (i.e., phenylacetic and phenylpropionic acids) are being developed as strong, non-invasive clinical biomarkers of the ‘high microbial metabolizer’ phenotype. If high levels of these terminal metabolites are in urine or plasma after supplementation, it suggests that the host’s gut microbiota has the genetic repertoire and high-enough taxonomic strain diversity to degrade anthocyanins fully, which may be associated with improved clinical outcomes [77]. The presence of these compounds provides supporting evidence for the systemic transport of bioactive metabolites. To a large degree, the trend of using these metabolites as clinical markers represents a change from focusing on the total amount of anthocyanins consumed to examining the different types of microbial fermentation products that are produced from the same dietary intake. There is considerable individual variability in the health benefits seen with anthocyanin supplementation, likely due to the large diversity of microorganisms that reside in the gut [15]. An individual’s ability to functionally use microbes is what makes the type and amount of bioactive metabolites produced unique. For instance, two subjects who have very different microbial makeups will be able to convert the same dose of anthocyanins into very different levels of PCA or any other phenolic acid, which will then lead to very different physiological outcomes [56]. In addition to that, the initial gut composition, such as the ratio of *Firmicutes* to *Bacteroidetes*, also differs after the same intervention [78]. Consequently, identifying an individual’s ‘metabolizer phenotype’ is important when designing dietary recommendations or clinical trials. Therefore, when creating a diet recommendation or conducting a clinical trial, it is important to know a person’s metabolizer phenotype. A shift from standard dose to a person’s individual dose emphasizes the importance of the microbiome in understanding how well each individual will respond to anthocyanins [79].

Recent studies have contributed to a shift in understanding of how anthocyanins work. Instead of understanding them in terms of an antioxidant, they are more appropriately understood using the pro-drug model. In this model, the majority of the therapeutic effect of anthocyanins is due to the enzymatic activity of the gut microflora [21]. The gastrointestinal process of moving food through the digestive system, the selectivity of modulating the microbiome with particular prebiotics [15,37], and ultimately producing bioavailable low-molecular-weight products (catabolites) combine to represent complex, interconnected systems governing how these compounds are handled by the body and their clinical outcomes.

## 7. Rewriting the Rules of Systemic Health via Metabolite-Driven Modulation and Underlying Mechanism

A shift in understanding has emerged whereby the focus has shifted from using the direct antioxidant capacity of anthocyanins as the mechanism responsible for their health benefits to a more advanced understanding of their metabolism and how they go through many metabolic transformations [80,81]. The previous paradigms viewed the gut microbiota at best as a passive site of nutrient transit; however, now we know that it is an active metabolizing organ that plays a key role in the production of bioactive compounds in our body [82]. This change is necessary due to the inherent structural limitations of anthocyanins’ native form as large hydrophilic glycosides that cannot easily diffuse passively across the intestinal epithelium and can only be minimally absorbed in the upper gastrointestinal tract [37]. The systemic health effects are proposed to be mediated primarily by their microbiota-derived metabolites, which interact with the key cellular signaling pathways to regulate oxidative stress, inflammation, and metabolic homeostasis [83]. As a result, the therapeutic effectiveness of dietary anthocyanins is closely tied to the structural characteristics of the corresponding metabolites rather than to the corresponding glycoside derivatives (Table 4) [4].

**Figure 4:**
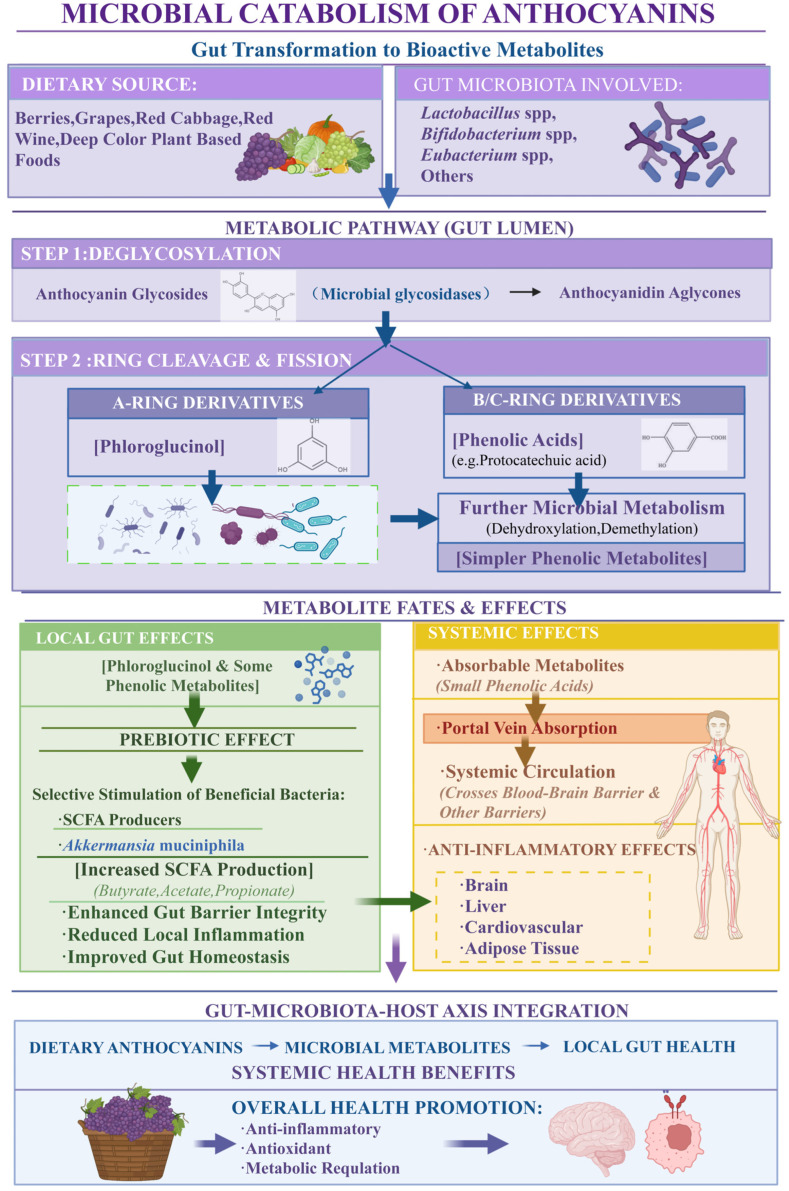
Microbial Metabolism of Anthocyanins and Interaction with Gut Microbiota. This figure illustrates the degradation of anthocyanins by gut microbiota, including deglycosylation and ring cleavage, leading to the formation of phenolic metabolites. It also depicts the interaction between these metabolites and gut microbial composition, as well as their transport into systemic circulation. Created in BioRender. Ahmed, M. (2026) https://BioRender.com/92mgpwk.

Recent research suggests that the key mechanism of action and biological effects are driven by these bioactive microbial metabolites rather than the original parent compounds, as depicted in the schematic illustration provided in Figure 4.

The <5% intact anthocyanins that are absorbed only account for a small fraction of total absorption in the human upper gastrointestinal tract (GIT). Overall, the gut’s ability to convert these compounds into their aglycones and then smaller phenolic metabolites increases their bioavailability and bioactivity [50,84]. These hybrid metabolites also improve the overall effectiveness of anthocyanins by increasing their bioavailability (summarized in Table 4). Aglycone-derived, specific phenolic metabolites with low molecular weights from anthocyanins can penetrate from systemic circulation into the CNS via the BBB, which appears to be responsible for several neuroprotective effects of these anthocyanin compounds [85]. This mechanism is a focus of recent studies on cognitive health improvement after anthocyanin intake [86]. Aglycone metabolites, such as protocatechuic acid and ferulic acid, are important regulators. They can activate the Nrf2 pathway, a master regulator of antioxidant enzymes and proteins, thereby mitigating oxidative stress in vivo. They also reduce inflammation through mechanisms that involve pathways such as NF-κB [37,56].

Aglycone metabolites directly contribute to anti-inflammatory and antioxidant effects within the gut itself [11]. As an example, catabolism of cyanidin-3-glucoside (C3G) in the gut produces metabolites that reduce inflammation and oxidative stress in the colon, optimizing conditions for metabolism [87]. These potent redox signaling modulatory properties may contribute to protection against chronic non-communicable and age-related pathologies, including cardiovascular disease, type 2 diabetes, certain cancers, and neurodegenerative disorders [88]. Supplementation with purified anthocyanin fractions attenuates the inflammatory response, as evidenced by the downregulation of key pro-inflammatory mediators such as CRP and IL-6 [36,43]. By modulating key redox-sensitive signaling pathways, anthocyanin-derived metabolites facilitate the restoration of cellular homeostasis, a process essential for mitigating exercise-induced oxidative stress and enhancing general health outcomes [4,59]. The generation of these metabolites begins with an initial degradative cleavage catalyzed by specific commensal taxa, such as *Bifidobacterium* spp. and *Lactobacillus* spp., which possess the requisite β-glucosidase activity [17,21]. Aglycone degradation and sequential metabolism are connected to the growth of these anaerobic beneficial bacteria. This effect is like a prebiotic, with anthocyanins and their relative metabolites acting as selective substrates that enhance beneficial taxa and modulate colonic microbial population ecology [18,56].

Additionally, anthocyanins in the diet help reshape the gut microbiome by stimulating the growth of distinct microbiota taxa known to produce short-chain fatty acids (SCFAs) such as *Faecalibacterium*, *Eubacterium*, *Roseburia*, and *Blautia* [21]. SCFAs (i.e., particularly butyrate, propionate, and acetate) play vital roles in maintaining the homeostasis of the intestine, specifically, lowering the luminal pH to prevent colonization of pathogens and help support the integrity of the intestinal barrier [89,90]. In addition to these metabolic effects, metabolites that come from the aglycone portion of anthocyanins also enhance the function of the intestinal barrier by promoting the expression of proteins in tight junctions and decreasing the permeability of the intestine, which is an important mechanism for reducing systemic inflammation caused by ‘leaky gut’ [5]. The structure of the anthocyanidin aglycone (e.g., cyanidin, delphinidin, or malvidin) influences the specific type and amount of metabolite produced, and this will therefore create a distinct shift in the microbiome [56]. There are opportunities to develop precision nutrition strategies, where standardized botanical extracts can then be used to grow specific microbiome patterns and potentially influence specific physiological responses (Figure 4). As more evidence emerges regarding the bioactive metabolites from anthocyanins, there is increasing interest in developing nutraceuticals and pharmaceuticals using these highly bioavailable phenolic metabolites and circumventing the poor absorption of the parent anthocyanins [37].

### 7.1. Intestinal Barrier Integrity and Anthocyanin-Derived Microbial Metabolites

The strengthening of intestinal epithelial barriers is one of the main local effects of consuming anthocyanins and is responsible for allowing many of the health benefits of anthocyanins, which are mediated by anthocyanin-derived microbial metabolites. The structural and functional preservation of the intestinal mucosal layer acts to decrease the likelihood of developing a ‘leaky gut’ and prevents the translocation of lipopolysaccharides (LPS) and other inflammatory microbial products into the systemic circulation [91]. Research indicates that anthocyanin intervention promotes intestinal morphology by increasing villus height and upregulating the expression of critical tight junction proteins such as occludin and zonula occludens-1. The key component of this process is targeting specific prebiotic stimulation of beneficial microbial populations in the gut [92,93].

*Akkermansia muciniphila* plays a vital role in maintaining barrier health through its ability to remodel the mucus layer as well as stimulate the host to produce additional mucin, thereby strengthening the physical barrier against luminal pathogens [92]. Anthocyanins provide health benefits due to a mechanism that does not involve direct systemic effects but rather initiates a cascade of events via their metabolism in the colon. The microbial conversion of anthocyanins to potent phenolic acids and short-chain fatty acids (SCFAs) results in the production of signaling molecules that regulate systemic and metabolic pathways, reduce neuroinflammation, and maintain immunological homeostasis in the barrier [94] (Figure 4). Therefore, this model shows that the gut ecosystem serves as an essential metabolic transducer, converting dietary anthocyanins into bioactive metabolites that ultimately determine their clinical utility [95].

### 7.2. Anti-Inflammatory Mechanisms of Anthocyanin-Derived Microbial Metabolite Profile

Anthocyanins can act as anti-inflammatory agents due to their ability to increase gut barrier function; their anti-inflammatory activity is primarily mediated by transforming microbes in the intestines and modulating the gut microbiome [96]. The major mechanism of action of the phenolic metabolites produced from anthocyanins is through inhibiting NF-κB, a critical transcriptional regulator of most genes involved with the development of pro-inflammatory response, resulting in reduced production of major pro-inflammatory cytokines (i.e., TNF-α and IL-6) [97,98]. Evidence from studies on animal models and in vitro systems demonstrates that PCA has an important role systemically, including its mediating role in anti-inflammatory properties. PCA attenuates macrophage NF-κB signaling, which is a mechanism by which systemic inflammation is reduced, leading to lower levels of pro-inflammatory cytokines (TNF-α, IL-6) [43]. Other phenolic acids generated through B-ring degradation (particularly gallic acid from delphinidins and syringic acids from malvidins) also exhibit strong anti-inflammatory effects and provide health benefits throughout the body independent of the parent anthocyanins [99]. Furthermore, gut microbial fermentation of anthocyanins markedly increases the concentration of SCFA (acetate, propionate, and butyrate) present in the lumen of the colon [95], which in turn reduces the colonic pH, leading to a less favorable environment for the proliferation of opportunistic pathogens.

Anthocyanin metabolites inhibit the growth of inflammatory bacterial populations, thereby decreasing the intestinal content of large amounts of lipopolysaccharides (LPSs), curtailing the trigger for systemic endotoxemia and chronic inflammation [100]. Although there may be variability among clinical manifestations of these molecular changes when examined in humans, their consistent dampening of inflammatory signaling markers confirms the therapeutic implications associated with this pathway [101]. Collectively, these anti-inflammatory effects illustrate that anthocyanin-derived metabolites have systemic modulating activity and convey very significant physiological protection via a mechanism in which the microbial fermentation products of dietary polyphenols have been translated to human physiology [102].

### 7.3. Impact of Anthocyanin-Derived Microbial Metabolite Profile on Hepatic Health

Anthocyanins and their microbiome-derived metabolites contribute to hepatic health by modifying lipid metabolism and slowing the development of NAFLD [5,103]. After moving into the liver through the portal circulation, specifically SCFAs and phenolic acids will inhibit the process of hepatic de novo lipogenesis [104], the major metabolic contributor to excessive intrahepatic lipid buildup. Additionally, by downregulating transcription factors involved in lipogenesis, such as SREBP-1c and FAS, these metabolites alter the hepatic environment to promote fatty acid β-oxidation rather than triglyceride synthesis [105].

These metabolites not only prevent the production of lipids but also protect cells against inflammation from long-term low levels of inflammation and oxidative stress associated with nonalcoholic fatty liver disease (NAFLD) [102]. Additionally, they play an important role in regulating cellular signaling in the liver by activating AMP-activated protein kinase (AMPK) to enhance mitochondrial function and restore hepatic energy balance [106]. These metabolites help to reduce the amount of pro-inflammatory bacterial lipopolysaccharides (LPSs) in gut muscle by enhancing the intestinal barrier, and thus help to suppress activation of the liver’s Kuffer cells [87]. The combined effect of both metabolic reprogramming and the reduction in inflammation has therapeutic value in preventing fatty liver disease caused by anthocyanin-derived microbial metabolites and reducing the likelihood that fatty liver will develop into a more serious type of metabolic liver disease [107].

### 7.4. Role of Anthocyanin-Derived Microbial Metabolite Profile in Cardiometabolic Health

Evidence indicates a close association between anthocyanins and their microbial metabolites with decreased risk of chronic diseases (cardiovascular disease, type 2 diabetes, and obesity) [108]. The protective mechanism begins with prebiotics like anthocyanins and the dietary fiber consumed together with them, because they change the composition of gut microbiota. One change is lowering the *Firmicutes*-to-*Bacteroidetes* ratio, which has been widely reported in individuals with metabolic dysfunction [42,109]. Modulation of gut microbiota composition stimulates increased production of short-chain fatty acids (SCFAs), acetate, propionate, and butyrate [18,19]. While butyrate predominantly provides energy for colonocytes to maintain gut barrier function, acetate and propionate enter the bloodstream to regulate peripheral lipid metabolism and peripheral glucose metabolism [95].

Beyond their role in metabolic regulation, these systemic metabolites modulate cellular pathways such as the activation of AMP-activated protein kinase (AMPK), which is a key to maintaining energy homeostasis and lipid synthesis [106]. Furthermore, these circulating metabolites exert direct vascular effects by enhancing endothelial function. By suppressing systemic inflammation and modulating nitric oxide (NO) bioavailability, these metabolites mitigate key physiological precursors of atherosclerosis [110]. Syringic acid may also benefit blood pressure management by improving the availability of nitric oxide (NO) to enhance the endothelial function of blood vessels and maintain homeostasis within vascular systems [110]. This dual action improves gut-derived metabolic signaling and directly protects the vascular integrity, which highlights the therapeutic potential of anthocyanin-mediated microbial metabolites in cardiometabolic health (Table 4).

### 7.5. Impact of Anthocyanin-Derived Microbial Metabolite Profile Along the Gut–Brain Axis

Emerging research has solidified the role of the gut–brain axis in mediating the neuroprotective effects of anthocyanins, positioning these compounds as potential interventions for the prevention and treatment of neurodegenerative diseases and central nervous system (CNS) injuries [17,111]. This process begins with the metabolic conversion of anthocyanins into bioactive phenolic metabolites, which can cross the blood–brain barrier, where they exert direct neuroprotective effects by suppressing microglial activation, a primary driver of chronic neuroinflammation in various neurological disorders [63]. Upon entering the CNS, PCA suppresses microglial activation to reduce chronic neuroinflammation and, thus, has significant neuroprotective properties [85].

Concurrently, the microbial production of short-chain fatty acids (SCFAs), stimulated by anthocyanin intake, serves as a vital systemic communication pathway. Butyrate in particular upregulates the expression of brain-derived neurotrophic factor (BDNF), which has an essential role in synaptic plasticity, memory, and neuronal survival [18,19]. Furthermore, these gut-derived signals modulate vagal nerve activity and reduce the concentration of circulating pro-inflammatory cytokines. By integrating these anti-inflammatory metabolites and SCFA-mediated signaling, the gut–brain axis functions as a critical regulator that links intestinal health to cognitive and emotional homeostasis [112].

## 8. Evidence Appraisal of the Microbiome–Metabolite Axis

To better understand the strength of evidence and limitations of available data, the evidence was also appraised using an evidence framework based on the GRADE approach, using the microbiome–metabolite axis as an example.

Anthocyanins, which interact with gut microbiota in a bidirectional manner, can modulate the microbial composition and diversity to enhance the growth of beneficial microorganisms such as *Bifidobacterium* and *Lactobacillus* species, and increase overall gut microbiota diversity [111,113,114]. Anthocyanins not only have nutritional values as supplements but may also have a prebiotic effect by being used as substrates for microbial fermentation for the establishment of a healthy gut microbiota [115]. Anthocyanins from *Lycium ruthenicum Murray* improved gut dysbiosis in mice through the promotion of beneficial *Lactobacillus* proliferation and improvement of intestinal barrier function [111]. Anthocyanins from blueberry and blackberry modulated gut microbiota and affected the metabolism of short-chain fatty acids to prevent metabolic syndrome in mice on a high-fat diet [113]. While human health effects of anthocyanins are mainly exerted through their metabolites produced by the human microbiome, Section 6 discusses potential health effects.

### 8.1. Methodological Limitations and Translational Gap

While evidence from in vitro and animal studies offers mechanistic insights, a gap in clinical evidence remains, with further studies required to confirm causal associations and translate to human-based studies.

#### 8.1.1. In Vitro Models

Anthocyanins and their intestinal metabolites have been studied in vitro by fecal fermentation. In vitro fecal fermentation by human gut microbiota transformed anthocyanins into several new metabolites with remarkable antioxidant activity [116,117]. Although an in vitro system provides valuable mechanistic insights into the metabolism by in vitro human gut microbiota—for example, in vitro fermentation of cyanidin-3-glucoside (C3G) increased the content of several metabolites and enhanced antioxidant properties [118]—the system has limitations, because in vitro models do not reflect several complex aspects of the human gastrointestinal tract, such as bioavailability, distribution, metabolism, and effects (BDMAs), and dynamic host–microbe interactions.

#### 8.1.2. Animal Models

Previous studies with animal models have suggested that anthocyanins, as well as gut microbiota, have beneficial health effects [111,119]. Previous studies investigated the effect of anthocyanin supplementation on gut microbiota in mice on a high-fat diet. Supplementation of high-fat diet-fed mice with anthocyanins elevated intestinal IgA production, altered gut bacterial community structure, improved liver steatosis, and suppressed obesity [113,119,120]. Findings from such animal experiments have limited external validity due to differences in gut microbiota, diet, and metabolism among animal models and human subjects. Although total conversion of a germ-free animal into a human microbial profile is feasible, reconstruction of the human microbial community composition in the murine gut is only partial.

#### 8.1.3. Human Clinical Trials

Human studies on anthocyanins include mainly observational epidemiological studies and an emerging number of clinical trials. Observational studies reported positive associations between consumption of anthocyanins and reduced risk of chronic diseases such as heart disease, various types of cancer, diabetes, and age-related cognitive impairment [76,121,122]. In clinical trials, supplementation with anthocyanins reduced inflammation and improved human metabolism-related parameters [121]. However, there are many limitations in the human studies, including differences in dietary anthocyanin sources, bioavailability, and inter-individual variations in gut microbiota composition and phenotypic metabolism [117]. While in vitro and in vivo studies suggested that some microbial-derived anthocyanin metabolites may have mechanistic roles, most human studies lack intervention-based evidence of health-promoting effects conferred by these microbial-derived anthocyanin metabolites. Outcomes can vary due to the human microbiome, genetics, and lifestyle.

### 8.2. Evidence Appraisal Framework (GRADE Equivalent Assessment)

Applying the GRADE framework (Grading of Recommendations Assessment, Development and Evaluation) reveals varying levels of evidence for different aspects of the anthocyanin–microbiome–metabolite axis:

#### 8.2.1. Relatively High-Certainty Evidence (Primarily from Preclinical and Mechanistic Studies)

Metabolism of Anthocyanins by Gut Microbiota:

The evidence (using in vitro studies and some human fecal evaluations) provides consistent evidence that the gut bacteria metabolize anthocyanins to produce a range of phenolic acids and other small compounds [26,116,117], and this occurs consistently for a wide range of types of anthocyanins and across different microbial communities.

Modulation of Gut Microbiota Composition:

Human intervention studies have found that consumption of anthocyanins has a beneficial effect on the gut microbiota by increasing numbers of beneficial species such as *Bifidobacterium* and *Lactobacillus*, and increasing the diversity of gut microbiota [111,113,123].

#### 8.2.2. Moderate-Certainty Evidence

Impact on Inflammatory Markers and Oxidative Stress:

In addition to preclinical models of disease suggesting possible anti-inflammatory effects, several human clinical studies show that anthocyanin consumption decreases markers of inflammation and oxidative stress [124,125]. While evidence supports the possibility that microbial-derived anthocyanin metabolites contribute to these observed effects, indirect evidence supports the direct effect of specific microbial-derived metabolites lowering human inflammation.

Improvements in Metabolic Parameters (e.g., Glucose, Lipids):

Considering that human trials and clinical studies indicate that anthocyanins may have beneficial health effects on several markers related to type 2 diabetes and metabolic syndrome [122,126], there may be a potential role for the microbial-derived metabolites identified in animal studies, which may be responsible in part for such health benefits in humans [113]. It will be important to monitor these bioactive metabolites, as well as the clinical markers related to diabetes and metabolic syndrome, in future trials.

#### 8.2.3. Low- to Very Low-Certainty Evidence (Plausible Hypotheses from Experimental Models)

Direct Causal Role of Specific Microbial Metabolites in Host Health:

Microbial metabolites of anthocyanins, such as the well-characterized short-chain fatty acids (SCFAs) associated with gut health benefits, have been studied extensively. However, for individual compounds, it is very challenging to make links between specific compounds and specific health effects, even after targeted intervention studies in humans. Much of our current understanding of their mechanisms of action still derives from in vitro and animal studies [127,128,129].

Neuroprotective Mechanisms via Gut–Brain Axis:

Studies on the interactions of anthocyanin metabolites with the gut–brain axis and their neuroprotective or cognitive effects have been conducted in animals [111,127,130]. Human studies on blueberries and related compounds have been primarily observational or large dietary trials, lacking insight into the active molecules.

Strengthening Intestinal Barrier Function:

Previous studies demonstrated that anthocyanins enhanced intestinal barrier function in animal models [5,111], and it has been proposed that their bioactive metabolites may exert similar effects on the human intestinal barrier. However, no study has identified specific anthocyanin metabolites capable of reinforcing or repairing the human intestinal barrier. Even though there is robust preclinical evidence for the biological activities of the microbiome–metabolite axis for anthocyanins, translation to human health needs to be further explored in clinical studies. For such studies on anthocyanins, it will be important to monitor individual anthocyanin metabolites, their bioavailability, and their different kinetic profiles, as well as their specific effects on biomarkers and clinical endpoints. To address this translational challenge, advanced multi-omics technologies and causal reasoning approaches will be needed [131]. While numerous studies have associated consumption of anthocyanin-rich foods and bioactive dietary compounds with health effects in humans, insight into the specific effects of individual metabolites needs to be gained to fully exploit the health-promoting axis.

## 9. Implication and Future Direction

The current evidence suggests that the gut microbiome functions as a mediator of anthocyanin bioactivity. Therefore, anthocyanins should not just be considered an antioxidant/end point substance, but rather the precursor to powerful metabolites/bioactive substances. The realization that the gut microbiome is the main regulator of anthocyanins may require a reassessment of designing clinical trials, developing nutrition guidelines, and creating the next generation of dietary interventions. Central to this is the urgent transition toward personalized nutrition [132]. The high inter-individual variability in response to anthocyanin supplementation, leading to inconsistent health outcomes, is primarily driven by differences in the host microbial enterotype [133]. Consequently, future therapeutic efficacy will likely depend on pre-screening an individual’s gut microbiome to predict metabolic capacity. Without specific microbial populations capable of performing essential biotransformation, such as deglycosylation and ring fission, the production of beneficial metabolites like protocatechuic acid (PCA) is significantly impaired, potentially limiting the effectiveness of the intervention [56]. Diagnostic advancement, such as fecal metagenomic sequencing or specialized metabolic breath tests, is therefore essential to define an individual’s metabolizer phenotype prior to intervention [134].

Current research is shifting toward the identification and isolation of specific bacterial strains, such as specialized *Bifidobacterium* or *Lactobacillus* species, capable of reliably metabolizing anthocyanin structures into desirable bioactive metabolites [135]. In order to provide maximum therapeutic predictability, researchers create rapid conversion to the aglycone and the resultant phenolic acid derivatives by combining anthocyanin glycosides with probiotics with high levels of β-glucosidase activity [136]. As a result, the current pharmacological definition of bioavailability, which is based upon systemic absorption of parent compounds, is inadequate in the case of anthocyanins since the concentrations of intact parent glycosides in plasma are often very low [40,43]. In this regard, it may be necessary to reexamine this definition and an alternative metric be developed, possibly using a ‘Microbial Bioactivity Index’ to reflect the cumulative and constantly evolving process of microbial metabolites, not merely the concentration of parent molecules within the bloodstream [137].

Ultimately, it is important that these interventions have sustainability in the long-term. Currently, most studies are either very short-term (4–12 weeks) or only evaluate a small number of patients, leading to significant uncertainty regarding the permanence of changes to microbiota, including increases in *Akkermansia muciniphila* or decreases in the F/B ratio. There are no longitudinal studies examining whether the benefits of taking dietary polyphenol supplements will continue to be seen after stopping supplementation or whether ongoing consumption is necessary for lasting effects. Furthermore, since most polyphenols in the diet occur as part of a complex whole-food matrix, future studies may need to study the interactions between classes of polyphenols (e.g., anthocyanins, proanthocyanidins, and lignans) to determine their combined effect on the final metabolite profile and associated health outcomes [40,56].

## 10. Conclusions

The evidence suggests that the concept of anthocyanin research has evolved from primarily looking at the transient, direct antioxidant activities of the parent compounds to a greater understanding of the microbiome–metabolite axis. According to our analysis, the gut microbiome appears to be a key intermediary that converts dietary anthocyanin consumption into robust, systemic health benefits. As a complex biotransformation engine, the gut microbiota is responsible for converting poorly absorbed anthocyanins into highly bioavailable, systemically active phenolic metabolites while facilitating the production of short-chain fatty acids that may act as important systemic effectors activating key anti-inflammatory, neuroprotective, and cardiometabolic signaling pathways. The transition from a ‘direct antioxidant activity’ approach to a microbiota-mediated health model highlights the potential importance of personalized approaches to nutrition, supporting the development of precision nutrition strategies by creating highly predictable, personalized dietary interventions that utilize modulation of the gut microbial ecosystem to help combat the increasing prevalence of chronic diseases.

## Figures and Tables

**Table 1:** Comparative Structural and Coloring Analysis of 4 Major Anthocyanins.

Name	R1 (3′)	R2 (5′)	Typical Color
Cyanidin	−OH	−H	Magenta/crimson
Delphinidin	−OH	−OH	Purple/blue
Pelargonidin	−H	−H	Orange/red
Malvidin	−OCH_3_	−OCH3	Purple

**Table 2:** Comparative Characteristic Analysis of SAR of Anthocyanin Based on R-1 and R-3.

Substitution Site	Common Sugars/Acids	Impact on Stability & Solubility
R3 (mono-glycosylation)	Glucose, galactose, arabinose	Baseline stability: It changes an unstable aglycone into a stable color pigment. It makes it more soluble in water.
R3 + R5 (Di-glycosylation)	Glucose–glucose	Enhanced Stability: By adding a second sugar at the fifth position, it protects the anthocyanin from degrading through pH bleaching.
Sugar + acyl group	p-Coumaric, ferulic, malonic acid	Maximum Stability: Some have referred to these compounds as acylated anthocyanins. They are also more stable against heat and light compared to other anthocyanins, which is why purple cabbage stays purple after being cooked.

**Table 3:** Key Anthocyanidin Aglycones, Their Microbial Degradation Metabolites, and Systemic Health Relevance.

Parent Aglycone	Chemical Structure	Key Degradation Metabolites	Chemical Structure	Systemic Importance (Usage)	References
Cyanidin	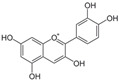	Protocatechuic acid (PCA), phloroglucinaldehyde	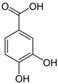 PCA	Strong antioxidant and anti-inflammatory activity; key role in neuroprotection by crossing the blood–brain barrier.	[36,43,44]
Delphinidin	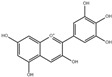	Gallic acid and syringic acid	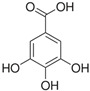 Gallic acid 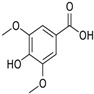 Syringic acid	Potent radical scavenging activity; associated with improved endothelial function and vasodilation	[45,46]
Malvidin (Mal)	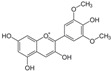	Vanillic acid, syringic acid and ferulic acid	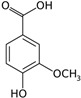 Vanillic acid 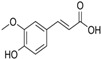 Ferulic acid	Improved lipid and cardiovascular health due to very stable and systemically absorbed anthocyanin metabolites	[43,47,48]
Peonidin/petunidin	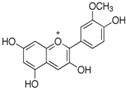	Vanillic acid	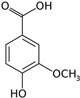 Vanillic acid	Known for cardioprotective effects and mitigation of oxidative stress in endothelial cells	[35]

**Table 4:** Key Health Effects and Proposed Mechanisms of Anthocyanin Metabolites.

Health System	Key Anthocyanin Metabolites	Primary Mechanism (Molecular Pathway)	Resulting Health Effect/Outcome	References
Anti-inflammatory Effects	Phenolic acids (e.g., PCA), SCFAs	Direct interference and suppression of NF-kappa beta signaling pathway. Local SCFA production leads to reduced gut pH.	↓ Cytokines, ↓ TNF-α, IL-6, ↓ systemic inflammation and LPS load.	[65,69]
Intestinal barrier function	Butyrate, microbial prebiotic effect	SCFAs (butyrate) act as fuel for colonocytes. Selective growth of *Akkermansia muciniphila*.	↑ Epithelial integrity and tight junction proteins; ↑ mucin production.	[83,84,85]
Cardiometabolic health	SCFAs (acetate, propionate), phenolic acids	SCFA signaling and microbial shift ↓ F/B ratio) leading to AMPK activation (energy homeostasis).	Regulation of lipid and glucose metabolism; improved endothelial function ↑ nitric oxide bioavailability.	[67,70,86]
Neuroprotection (gut–brain axis)	Phenolic acids (e.g., PCA), butyrate	Phenolic metabolites cross the blood–brain barrier (BBB), systemic SCFAs and metabolites modulate the vagal nerve.	↓ Neuroinflammation; (↓ microglial activation); ↑ brain-derived neurotrophic factor (BDNF) expression (synaptic health).	[81,82,87]

## Data Availability

No new data were created or analyzed in this study.
